# Integrating a Smart Sensor Chip and AI Predictive Analytics Into the Sehhaty App to Enhance Diabetes Management in Saudi Arabia

**DOI:** 10.7759/cureus.104417

**Published:** 2026-02-27

**Authors:** Abdullah F ALqunisi

**Affiliations:** 1 Health and Rehabilitation Sciences, University of Pittsburgh, Pittsburgh, USA

**Keywords:** diabetes management, mobile health, predictive analytics, saudi arabia, sehhaty app, smart sensor chip

## Abstract

Diabetes is a major public health challenge in the Kingdom of Saudi Arabia. A substantial proportion of adults are affected, placing significant pressure on the healthcare system. Although digital health initiatives have expanded in recent years, patients continue to encounter barriers to adopting mobile health (mHealth) technologies, including technical limitations, usability concerns, and privacy issues.

This article proposes a comprehensive digital health solution for diabetes management that enhances the national health application Sehhaty by integrating two complementary technologies: a smart sensor chip (SSC) for continuous physiological monitoring and AI-based predictive analytics (AIPA) for forecasting glycemic trends. The aim is to strengthen proactive and personalized diabetes care.

An Agile development framework consisting of two sprints is proposed. The first sprint integrates a wearable SSC into the Sehhaty ecosystem to transmit real-time glucose and vital sign data through secure wireless communication. The second sprint develops AIPA using machine-learning models to analyze data patterns and predict glycemic fluctuations. System requirements were identified through stakeholder engagement, and the architecture includes a secure cloud backend, structured data flow, and user-centered interface design.

The integration of SSC and AIPA into Sehhaty could enhance diabetes management by enabling continuous monitoring, personalized alerts, and earlier intervention. The system design prioritizes reliability, user-centered usability, and data privacy safeguards to address common barriers to digital health adoption. Consideration of perceived usefulness, ease of use, trust, and accessibility informed the development strategy.

The proposed SSC-AIPA framework has the potential to transform Sehhaty into an advanced diabetes management platform that supports early detection, predictive insights, and individualized care. Future work will include prototyping, usability evaluation, and clinical validation.

## Introduction

Diabetes mellitus is a chronic metabolic disorder characterised by persistent hyperglycaemia and long-term microvascular and macrovascular complications. The disease has reached epidemic proportions in the Kingdom of Saudi Arabia, placing substantial pressure on the national healthcare system. According to recent epidemiological analyses, diabetes prevalence in Saudi Arabia remains among the highest globally, with marked regional and demographic disparities across the population [1]. Socio-economic assessments further demonstrate significant variation in prevalence rates between urban and rural communities, as well as across different educational and age groups [2]. These findings highlight the urgent need for scalable, technology-enabled interventions to support prevention, monitoring, and long-term disease management.

In response to the growing burden of chronic diseases, the Saudi Ministry of Health has implemented Sehhaty, a national mobile health (mHealth) application designed to provide appointment scheduling, vaccination records, and access to personal health information. However, current mHealth platforms often face technical and usability barriers, including limited device integration, privacy concerns, and restricted functionality, which may hinder sustained adoption [3]. To address these limitations, this technical report proposes the integration of a smart sensor chip (SSC) with AI predictive analytics (AIPA) within the Sehhaty ecosystem. The proposed solution aims to enhance continuous glucose monitoring, improve clinical decision support, and strengthen user engagement through secure, data-driven personalised feedback.

This study presents a technical proposal and conceptual framework for integrating a smart sensor chip (SSC) with artificial intelligence predictive analytics (AIPA) into the Sehhaty platform. The purpose of this work is to describe the system architecture, anticipated workflow, and potential clinical impact rather than report empirical validation results. The proposed framework is intended to support future pilot testing and clinical evaluation phases.

## Technical report

This technical report outlines the design and development of an integrated digital health solution aimed at enhancing diabetes management within the Sehhaty application. The Agile methodology was adopted to support iterative development, stakeholder feedback, and continuous refinement. The project is structured into two sequential sprints to ensure systematic implementation and validation. The smart sensor chip (SSC) described in this framework represents a conceptual hardware integration model rather than a fully validated commercial device. Design considerations include calibration accuracy, signal reliability, and interoperability with electronic health record (EHR) systems using standardized communication protocols such as Health Level 7 (HL7) and Fast Healthcare Interoperability Resources (FHIR).

Sprint 1 - smart sensor chip (SSC) integration

The first sprint focused on integrating a smart sensor chip with the Sehhaty platform. Stakeholder workshops involving representatives from the Ministry of Health, clinicians, and diabetic patients were conducted to define system requirements, including key physiological parameters (glucose, heart rate, and respiration), measurement frequency, and device integration constraints. A conceptual data flow diagram was developed to illustrate interoperability between the smart sensor chip, the Sehhaty mobile application, and a secure cloud-based backend. The architecture specifies encrypted data transmission via Bluetooth or 5G and adherence to national data protection regulations. Development activities centred on integrating a commercially available continuous glucose monitoring device into Sehhaty through an application programming interface (API), alongside implementing modules for device pairing, real-time data acquisition, and secure storage. Deployment involved pilot testing with a small patient cohort to evaluate data accuracy, connectivity stability, and user satisfaction, with findings informing iterative system enhancements.

Sprint 2 - AI predictive analytics (AIPA) development

The second sprint concentrated on developing AI-based predictive analytics to forecast glycaemic excursions. System requirements included the collection of anonymised longitudinal datasets comprising glucose readings, heart rate metrics, activity patterns, and user interaction logs. The design phase defined machine-learning workflows and selected appropriate time-series prediction models, such as long short-term memory (LSTM) networks. Cloud-based AI services (e.g., Microsoft Azure AI) were utilised for model development, training, and validation using historical data. A dedicated user interface was designed to visualise predictive outputs and personalised recommendations. During deployment, the AIPA module was integrated into Sehhaty to generate real-time alerts and tailored guidance, with performance evaluation conducted through structured user testing. Long short-term memory (LSTM) networks were selected due to their effectiveness in modeling temporal physiological data sequences characteristic of glucose variability patterns. Alternative architectures such as transformer-based models were considered; however, LSTM provides an appropriate balance between computational efficiency and predictive performance for longitudinal health data. Potential risks of algorithmic bias related to demographic variability and data imbalance were considered, and model explainability approaches are proposed to enhance clinical interpretability.

Model validation is planned using standard performance evaluation metrics, including area under the curve (AUC), root mean square error (RMSE), prediction accuracy, sensitivity, and specificity. Cross-validation techniques and periodic model retraining strategies are proposed to maintain model reliability over time. At the current stage, pilot testing is conceptual and simulated based on anticipated workflows rather than involving real-world participants; future implementation phases will include user-based clinical validation.

Data flow

**Figure 1:**
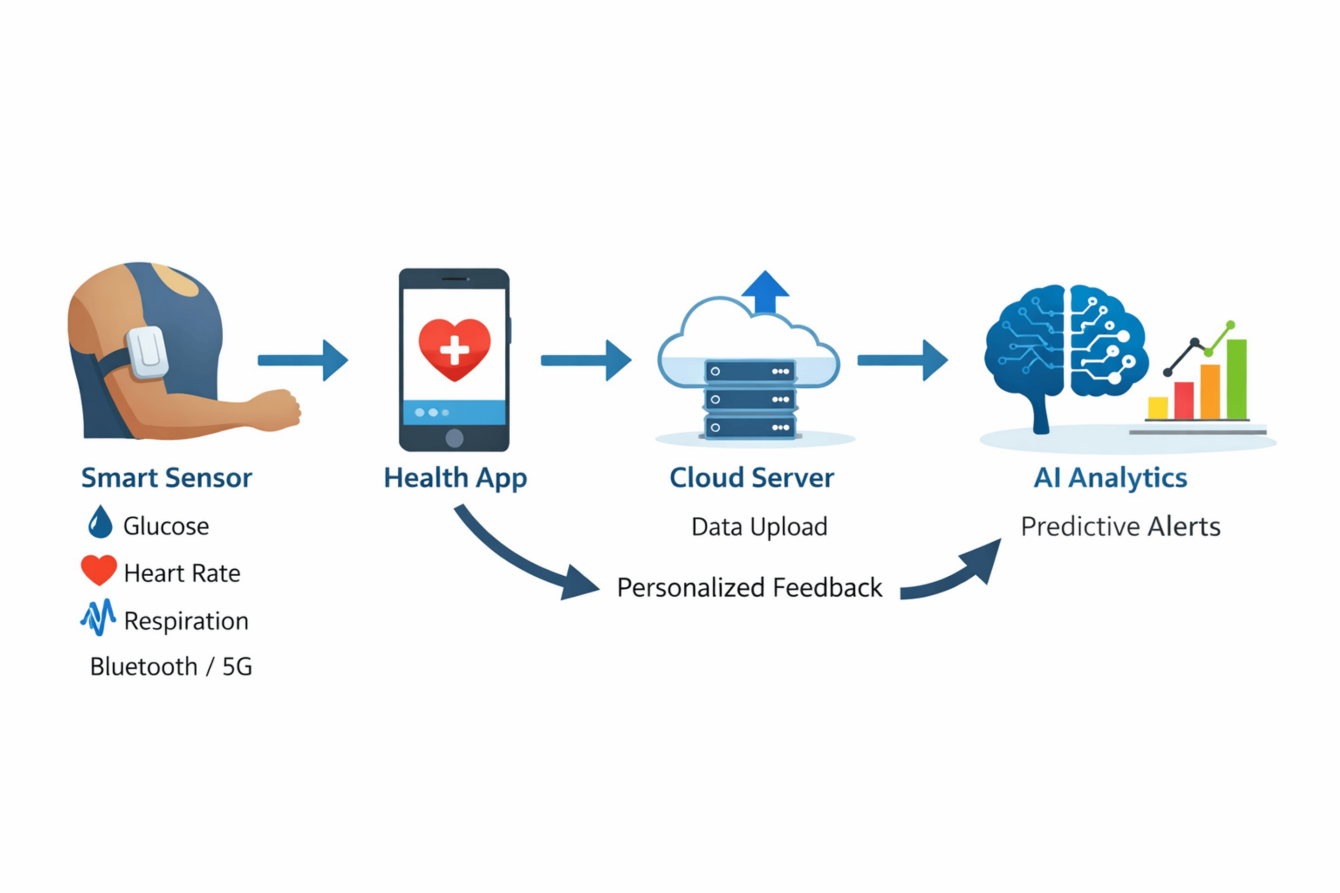
Conceptual data flow of the smart sensor chip-AI predictive analytics integration with the Sehhaty app A smart sensor chip (SSC) continuously measures glucose, heart rate, and respiration and transmits the data via Bluetooth or 5G to the patient's smartphone running the Sehhaty app. The app uploads data securely to a cloud server where AI predictive analytics (AIPA) models analyse trends and forecast glycaemic excursions. The server returns personalised alerts and recommendations to the app for patient action Figure created by the author.

The smart sensor chip continuously collects physiological data and transmits it to the patient's smartphone via Bluetooth or 5G. The Sehhaty application displays the readings, securely stores them within a cloud database, and enables controlled data sharing with authorised healthcare providers when required. The AI predictive analytics module analyses longitudinal trends to detect patterns and predict future glucose fluctuations. Based on these predictions, patients receive actionable alerts and personalised recommendations through Sehhaty, facilitating timely clinical and behavioural interventions. The overall system architecture and data flow of the proposed SSC-AIPA integration are illustrated in Figure 1. 

A phased implementation roadmap is proposed, beginning with pilot testing, followed by regional deployment, and eventual national-scale integration contingent upon successful validation outcomes. Measurable evaluation endpoints will include predictive accuracy, system latency, user satisfaction scores, and clinical outcome indicators such as HbA1c trends and hypoglycemic event reduction.

The system architecture incorporates multiple security layers, including encryption protocols, authentication checkpoints, and secure cloud storage mechanisms to ensure patient data confidentiality and compliance with national health data protection regulations.

## Discussion

Recent research emphasises that technical malfunctions, poor usability, privacy concerns, and limited functionality remain significant barriers to the adoption of the Sehhaty app [2]. The proposed SSC-AIPA integration addresses these limitations by prioritising system reliability, user-centred design principles, and robust privacy safeguards. Studies grounded in the Technology Acceptance Model (TAM) further indicate that perceived usefulness, ease of use, privacy, and trust strongly influence patients' intention to adopt digital health services [2]. By enabling continuous monitoring, personalised alerts and secure data management, the SSC-AIPA system enhances both perceived usefulness and user trust.

Existing continuous glucose monitoring (CGM) integrations within national digital health applications often lack predictive intelligence and real-time clinical decision support capabilities. International digital health initiatives increasingly incorporate AI-driven analytics to enhance chronic disease management; however, interoperability challenges, data governance concerns, and user adoption barriers remain. The proposed SSC-AIPA framework aims to address these technological and clinical gaps by enabling predictive insights alongside secure integration within the national health infrastructure.

Furthermore, socio-economic analyses suggest that diabetes prevalence is disproportionately higher among older adults, individuals with lower educational attainment and populations residing in rural areas [1]. To promote equitable access, the proposed framework leverages the extensive national coverage of Sehhaty while advocating for digital literacy initiatives and targeted device subsidy programmes. Potential implementation challenges-including sensor affordability, patient adherence, and data governance concerns-require continuous evaluation, stakeholder engagement, and structured user education strategies to ensure sustainable and equitable adoption.

Ethical governance considerations are central to the proposed system, including patient data privacy, algorithm transparency, accountability in clinical decision support, and mitigation of potential algorithmic bias. Cost-effectiveness is anticipated through improved disease control, reduction in acute complications, and decreased healthcare utilization; however, formal economic evaluation remains a subject for future research.

It is important to distinguish between anticipated benefits and evidence-based outcomes. While the conceptual framework demonstrates potential to enhance diabetes management through predictive analytics, empirical validation through pilot studies and real-world implementation will be required to confirm clinical effectiveness and long-term impact.

## Conclusions

Diabetes imposes a substantial burden on Saudi Arabia's healthcare system, with prevalence rates among the highest globally. While the Sehhaty platform provides a foundation for digital health engagement, current functionality lacks continuous monitoring and predictive capabilities. The proposed integration of a smart sensor chip with AI predictive analytics represents a conceptual framework with the potential to enhance diabetes management through personalized alerts, improved self-management support, and strengthened clinical decision-making.

However, the proposed system requires further technical development, pilot testing, and real-world clinical validation to confirm effectiveness, usability, and long-term impact. Future research should focus on large-scale implementation, performance evaluation, and economic assessment to determine the feasibility of national deployment.
